# A Review of 2009 for *PLoS Computational Biology*

**DOI:** 10.1371/journal.pcbi.1000687

**Published:** 2010-02-26

**Authors:** Rosemary Dickin, Cecy Marden, Catherine Nancarrow, Philip E. Bourne

**Affiliations:** 1 Department of Pharmacology, University of California San Diego, La Jolla, California, United States of America; 2 Skaggs School of Pharmacy and Pharmaceutical Sciences, University of California San Diego, La Jolla, California, United States of America

2009 was another strong year for *PLoS Computational Biology*. As in 2008, we saw growth and development at every turn—our submissions and publishing presence, the level of quality of the work we considered and published, the degree of community engagement with the journal, and our editorial leadership.

As we briefly review the year past and reveal plans for the year to come, one conclusion is undeniable: we are truly a community journal, the achievements of which result from and depend on the contributions of our authors, readers, editors, and reviewers. Our thanks to you all for your continued support, trust, and partnership in advancing the field in which we work.

*PLoS Computational Biology*'s growing position in the field is evidenced by the statistics for 2009. We recorded 1,204 research articles submitted to the journal—an increase of 31% from 2008. Of these submissions, we published a total of 344 (29%), along with 33 Reviews, Perspectives, and Education articles. Of the total full submissions, 41% were rejected without peer review. Such an increase in our submission volume prompted the addition of 11 new editors to our Editorial Board, the recruitment of 128 guest editors, and the support of 1,616 reviewers (see Table S1). And, importantly, our readership grew this year as well. The number of readers receiving our new article alerts now exceeds 14,000, and our press coverage worldwide alerted countless more to the high-quality science we have the privilege to publish.

By traditional measures comprising a variety of “factors,” whether impact, Eigen, or H, the journal is doing very well and represents an important open-access contributor to the field. There are now more important metrics, however, by which to measure the quality of a published paper: the usage statistics and other measures of community response now available at the article level. In the Fall of 2009, PLoS introduced article-level metrics across all journals, making it possible to see, among other metrics, the number of views and downloads each paper receives in real time. The “Metrics” tab on each article presents a summary of all activity post-publication, which has proven to be of great interest to the readers and authors alike.

Also available on our Web site is a summary Excel file of the journal's entire corpus (see http://www.ploscompbiol.org/static/journalStatistics.action#PLoSCompBiol), which provides the opportunity for some very interesting analyses. For example, some highly downloaded articles, like the Ten Simple Rules series, are not likely to be cited frequently but consistently draw readers. This brings the issue of scientific merit and reward sharply into focus, raising the question, What does indeed represent a scientific contribution? Taking a different view of the data, the average number of downloads for any article is over 2,000 with a strong showing in mathematically oriented articles and those relating to genetics and genomics. These divisions are based on author-provided keywords and tell only part of the story of what is “hot” in our field. The summary statistics show clearly that computational neuroscience continues to be a strong area of the journal with modeling of biological systems at various scales a definite sphere of growth. We encourage you to view the data and perform your own analyses—and let us know your results.

Our relationship with the International Society for Computational Biology (ISCB) remains strong. In 2009, we continued to publish Messages from ISCB on a variety of topics and, as in years past, contributed to the Society's annual meeting, Intelligent Systems for Molecular Biology (ISMB) 2009, in Stockholm, Sweden. This year, *PLoS Computational Biology* organized a special session on “Advances and Challenges in Computational Biology,” chaired by Deputy Editor Barbara Bryant. Three members of the journal's Editorial Board—Abigail Morrison, Adam Arkin, and Donna Slonim—highlighted recent scientific advances made possible by computation and mathematics in the respective fields of computational neuroscience, synthetic biology, and translational medicine in human development.

With 2009 behind us, we look forward to continued strength in another area of the journal—our non-research articles. To add to our popular Editorial, Education, and Review series, we will be introducing some exciting features in 2010 that we hope will appeal to our broad readership. An ongoing “Roots of Bioinformatics…” series, edited by David Searls, will provide insights into how various areas of the discipline developed. These will be personal perspectives from scientists who helped to shape the field and will be compelling and inspirational reading for those entering or thinking of entering this vibrant arena. A “Postcards from” feature, designed to capture the highlights of important computational biology conferences, will provide the opportunity for the younger members of our community to comment and offer a fresh perspective on new developments described in presentations and through dialogue. We welcome your feedback and ideas on this new content as the year progresses.

Another, and different, editorial goal in 2010 is to improve our service to our authors, particularly with regard to reducing the time to first decision for papers that we do not intend to consider for publication. Our 2009 records show an average interval of 45 days from submission to a first decision for peer-reviewed manuscripts—and we aim to do better. We are committed to this task, but are equally committed to ensuring that any steps we take do not compromise the quality of the review. To help us serve you better, we encourage the regular practice of submitting a presubmission inquiry rather than a full submission. This feature allows us to preview the paper and offer a far faster response, in a matter of days, as to the likely suitability of your paper for the journal. If you have other thoughts and comments on the state of the journal and what we should be doing—or doing better—we encourage you to use the commenting feature on an article that prompts your comment or, if you prefer, by contacting the editorial office directly at ploscompbiol[at]plos.org.

Thank you once again for your ongoing support. We wish you all well in your research endeavors during 2010.

## Supporting Information

Table S1 Guest Editors and Reviewers for *PLoS Computational Biology* in 2009.
(0.27 MB PDF)

