# Supplementary material for: A Review of 2009 for PLoS Computational Biology
Source: PLoS Comput Biol. 2010 Feb 26;6(2):e1000687. doi: 10.1371/journal.pcbi.1000687 (PMC2829024; doi:10.1371/journal.pcbi.1000687)
Supplement: Table S1 — Guest Editors and Reviewers for PLoS Computational Biology in 2009. (0.27 MB PDF) [file pcbi.1000687.s001.pdf]

PLoS Computational Biology Guest Editors 2009

Alizon, Samuel
Alter, Orly
Amos, Tanay
Asquith, Becca
Ast, Gil
Böckmann, Rainer A
Babu, Madan
Backx, Peter H.
Bader, Gary D
Balaji, Petety
Bar-Joseph, Ziv
Barrick, Doug
Beerenwinkel, Niko
Biham, Ofer
Breakspear, Michael
Burrage, Kevin
Bussemaker, Harmen J.
Bystroff, Chris
Carloni, Paolo
Case, David
Cheatham, Tomas E
Chechik, Gal
Cooke, Roger
Cui, Qiang
Darling, Aaron E.
Deane, Charlotte
Deco, Gustavo
Dokholyan, Nikolay V.
Elber, Ron
Elofsson, Arne
Feuk, Lars
Finn, Robert D.
Fletcher, Daniel A.
Floudas, Christodoulos
Garcia, Angel
Gardner, Paul P.
Gasch, Audrey Patricia
Gerstein, Mark
Ghani, Azra C
Gilson, Michael
Godzik, Adam
Goodhill, Geoffrey J.
Gottesman, Michael M.
Goulian, Mark

Gräter, Frauke
Hofacker, Ivo L
Horovitz, Amnon
Howard, Jonathon
Hummer, Gerhard
Huynen, Martijn A.
Ideker, Trey
Ioshikhes, Ilya
Jansen, Vincent A.A.
Jones, David
Kishony, Roy
Kitano, Hiroaki
Kolomeisky, Anatoly
Kortemme, Tanja
Kubler, Janet
Lahav, Galit
Lee, Doheon
Lemey, Philippe
Levine, Erel
Levy, Ronald
Liang, Jie
Lichtarge, Olivier
Loria, Patrick
Lowe, Todd
Maloney, Laurence T.
Maranas, Costas D.
Marrink, Siewert-Jan
McCluskey, Connell
Michor, Franziska
Mirny, Leonid A.
Mooney, Sean David
Morris, Quaid
Nowotny, Thomas
Ofran, Yanay
Onuchic, Jose
Pacheco, Jorge M.
Paulsson, Johan
Pelkmans, Lucas
Pettitt, B. Montgomery
Pfeiffer, Thomas
Pomerening, Joseph R.
Pries, Axel R
Prisk, Kim
Przytycka, Teresa Maria

Richardson, Jane
Richardson, Richard B.
Rohwer, Forest
Ruppin, Eytan
Scheres, Ben
Schuster, Stefan
Schwede, Torsten
Schwikowski, Benno
Serrano, Luis
Shatkay, Hagit
Sherman, Jamie
Shoichet, Brian
Shvartsman, Stanislav
Smith, Thomas A
Smolke, Christina D
Stitt, Mark
Sukharev, Sergei
Szabo, Gyorgy
Tajkhorshid, Emad
Tanaka, Mark M
Taylor, William
Thieffry, Denis
Tjaden, Brian
Unger, Ron
Valencia, Alfonso
van Nimwegen, Erik
Verkhivker, Gennady M
Verschure, Paul F.M.J.
Vishveshwara, Saraswathi
Wahl, Lindi
Walter, Nils
Wiest, Olaf G.
Wilke, Claus O.
Workman, Christopher
Xu, Dong
Xu, Ying
Zavolan, Mihaela
Zhang, Jianzhi
Zhou, Huan-Xiang
Zimmer, Christophe

PLoS Computational Biology Reviewers 2009

Abarbanel, Henry
Abate, Alessandro
Achard, Pablo
Achard, Sophie
Adami, Chris
Adams, Daniel
Adams, Paul
Adryan, Boris
Aebi, Ueli
Aerts, Stein
Agarwal, Pankaj
Aguda, Baltazar D.
Ahern, Chris
Akerman, Colin
Ala-Korpela, Mika
Alarcon, Tomas
Albert, Istvan
Albert, Reka
Albrecht, Mario
Aldridge, J. W.
Alexov, Emil
Alkan, Can
Allen, Charles
Allen, Timothy
Almaas, Eivind
Almonacid, Daniel E.
Alon, Uri
Aloy, Patrick
Alt, Wolfgang
Altan-Bonnet, Grégoire
Alter, Orly
Althaus, Christian L.
Altman, Russ B.
Altmann, Andre
Altschuler, Steven J.
Alves, Rui
Amaral, Luis A.
Amirikian, Bagrat
An, Gary
Ananiadou, Sophia
Andrade-Navarro, Miguel A.
Andreasen, Viggo
Andricioaei, Ioan
Andrzejak, Ralph
Antes, Iris
Antia, Rustom
Antoniewicz, Maciek
Aoki, Kenichi
Aoki-Kinoshita, Kiyoko F.
Aqvist, Johan
Arathorn, David
Arita, Masanori
Arkin, Isaiah
Armitage, Judith P.
Arnold, Jonathan
Arstila, T. Petteri
Asbury, Charles
Ashburner, John
Asquith, Becca
Ast, Gil
Atilgan, Canan
Aunger, Robert
Averbeck, Bruno
Azad, Rajeev K.
Böckmann, Rainer A.
Babbitt, Patricia C.
Bach, Michael
Backofen, Rolf
Backus, Benjamin T.
Bader, David
Bader, Gary D.
Bader, Joel S.
Baehler, Jurg
Bair, Wyeth
Bajaj, Chandra
Balduzzi, David
Ball, Catherine A.
Balloux, François
Balsa-Canto, Eva
Banga, Julio R.
Banks, Martin
Bansal, Shweta
Bar-Joseph, Ziv
Baraniuk, Richard
Barbas, Helen
Bardwell, James
Barik, Sailen
Barkai, Naama
Barth, Patrick
Bartol, Thomas M.
Bassett, Danielle
Bassingthwaighte, James
Basso, Luiz
Bassukas, Ioannis
Bastolla, Ugo
Bates, Paul A.
Battaglia, Francesco
Batzoglou, Serafim
Bauch, Chris T.
Baumbach, Jan
Baumketner, Andriy
Baxter, Douglas
Bazhenov, Maxim
Beauchemin, Catherine A.
Beck, Jeff
Becskei, Attila
Beek, Peter
Beer, Michael A.
Beerenwinkel, Niko
Behrens, Tim
Beissbarth, Tim
Bell, Douglas A.
Bell, George W.
Beltman, Joost
Beltrao, Pedro
Bendtsen, Jannick
Benfey, Philip N.
Benos, Panayiotis (Takis) V.
Benson, Gary
Benucci, Andrea
Berg, Howard C.
Berg, Johannes
Bergman, Casey M.
Bergman, Nicholas
Bergmann, Sven
Berkes, Pietro
Berman, Helen
Bernacchia, Alberto

Berridge, Kent C.
Berriman, Matthew
Bertonati, Claudia
Best, Robert
Beverley, Peter C.
Bex, Peter
Bhalla, Upinder S.
Bhatnagar, Aruni
Biek, Roman
Binzegger, Tom
Blake, Judith A.
Blanpied, Tom
Blaschke, Christian
Blencowe, Benjamin
Bogacz, Rafal
Bolker, Benjamin M.
Boni, Maciej F.
Bonneau, Richard
Boone, Charles
Boonman, Arjan
Bootsma, Martin
Borenstein, Elhanan
Borghans, Jose A.
Borisyuk, Alla
Bornberg-Bauer, Erich
Bornholdt, Stefan
Borst, Alexander
Bose, Indrani
Boshoff, Helena
Bourret, Robert B.
Boutros, Paul C.
Bowie, James U.
Bowman, Howard
Braga-Neto, Ulisses
Braun, Terry A.
Breakspear, Michael
Brehelin, Laurent
Brenner, Eli
Bressloff, Paul
Briggs, James M.
Brinkman, Fiona S.
Brody, Steven L.
Brooks, Charles L.
Brosius, Jürgen
Brown, Emery N.
Brown, James
Brudno, Michael
Brunel, Nicolas
Brusic, Vladimir
Bryc, Kasia
Bryson, Joanna
Bucher, Philipp
Buchler, Nicolas
Buckmaster, Paul
Budinger, G.R.Scott
Buettner, Garry R.
Buhler, Jeremy
Bullinger, Eric
Bullmore, Ed
Bullock, Daniel
Bulsara, Adi
Bulyk, Martha L.
Burgard, Anthony
Burgess, Neil
Burke, Dan
Burkitt, Anthony
Burns, Gully
Burr, David C.
Burrage, Kevin
Burton, Dennis R.
Bussemaker, Harmen J.
Butcher, Sarah
Butte, Nancy
Butts, Daniel A.
Butz, Markus
Bystroff, Chris
Caflisch, Amedeo
Cai, David
Calin, George
Callard, Robin
Calogero, Raffaele A.
Canavier, Carmen C.
Cannon, Robert C.
Cantley, Lewis C. C.
Carbone, Alessandra
Carloni, Paolo
Carlsson, Anders
Carpena, Pedro
Cartwright, Julyan H.
Carvunis, Anne-Ruxandra
Castellanos, F. Xavier
Cauchemez, Simon
Cavasotto, Claudio
Cellmer, Troy
Chadwick, Richard S.
Chahine, Mohamed
Chakraborty, Arup K. K.
Champagne, Frances
Chan, Christina
Chan, Hue-sun
Chandra, Nagasuma
Chang, Roger
Changeux, Jean-Pierre
Chappell, Michael
Chate, Hugues
Chaves, Madalena
Chechik, Gal
Chen, Jake Y.
Chen, Kevin
Chen, Yu zong
Chesler, Elissa
Cheung, Margaret
Cheung, Warren A.
Chialvo, Dante R.
Chitnis, Nakul
Chizhov, Anton
Chodera, John
Chou, Tom
Chuang, Trees-Juen
Chumbley, Justin
Churchill, Gary A.
Clancy, Colleen E.
Clark, Andrew G.
Cleland, Thomas
Clermont, Gilles
Clewley, Jonathan
Climer, Sharlee
Cline, Melissa
Colbert, Costa
Colijn, Caroline
Collins, James J.
Colman-Lerner, Alejandro
Colombo, Giorgio
Colquhoun, David
Connor, Charles E.

Coombes, Steve
Coombs, Dan
Cornish-Bowden, Athel
Cosentino Lagomarsino, Marco
Cossart, Rosa
Coutsias, Evangelos A.
Covert, Markus W.
Cox, Edward C.
Crampin, Edmund J.
Crauste, Fabien
Csermely, Peter
Csikasz-Nagy, Attila
Cuff, Alison
Cui, Qiang
Cuntz, Hermann
Dallon, John
Dandekar, Thomas
Dantas, Gautam
Darling, Aaron E.
Das, Debopriya
Das, Jayajit
Das, Rhiju
Dash, Ranjan
Daunizeau, Jean
Davenport, Miles P.
David, Olivier
David, Stephen
Davidich, Maria I.
Davis, Ian
Davuluri, Ramana
Daw, Nathaniel
Dayan, Peter
De Boer, Rob J.
De Groot, Bert L.
De Haan, Gerald
De Jong, Hidde
De Oliveira, Tulio
De Polavieja, Gonzalo
De Schutter, Erik
De Silva, Eric
De Visser, J. Arjan G. M.
De Vries, Gerda
Dean, Paul
Deber, Charles

Deco, Gustavo
DeFroment, Adrian
Degenhardt, Jeremiah D.
DeGroot, Marcel
Dekker, Job
Dell'Acqua, Flavio
Dell'Orco, Daniele
Deneve, Sophie
Denham, Susan L.
Dennis, Jonathan
Deo, Rahul C.
Dermitzakis, Emmanouil T.
Desai, Michael M.
Dessailly, Benoit H.
Destainville, Nicolas
Dewey, Colin N.
Dey, Sutirth
Di Cera, Enrico
Diamond, Scott L.
Diedrichsen, Jörn
Diesmann, Markus
Diggle, Peter J.
Ding, Li
Ding, Ye
Dingli, David
Dinner, Aaron R.
Djurfeldt, Mikael
Do, Chuong
Doebeli, Michael
Doering, Charles
Domany, Eytan
Domingo, Esteban
Domingues, Francisco S.
Domini, Fulvio
Dominy, Brian
Doms, Robert W.
Donchin, Opher
Doolittle, Russell F.
Dosztányi, Zsuzsanna
Doyle, Declan
Doyle, Frank
Draghici, Sorin
Drozdov, Ignat
Drummond, Allan
Dubchak, Inna L.

Dumontier, Michel
Dunbrack, Roland
Duneau, Jean-Pierre
Dunker, Keith
Dunne, Jennifer
Durand, Jean-Baptiste
Durell, Stewart
Durrens, Pascal
Durstewitz, Daniel
Dyson, Jane
Ebenhöh, Oliver
Eddy, Sean R.
Edelstein-Keshet, Leah
Edwards, Andy
Edwards, Nathan
Edwards, Richard J.
Einhaeuser, Wolfgang
Eisen, Jonathan A.
El Karoui, Meriem
Elber, Ron
Elemento, Olivier
Elf, Johan
Eliasmith, Chris
Elkon, Ran
Elofsson, Arne
Emanuelsson, Olof
Emery, Michael
Emonet, Thierry
Enderling, Heiko
Endres, Robert G.
Endy, Drew
Engström, Pär G.
Ermentrout, Bard
Ernst, Jason
Ernst, Marc
Ernst, Udo A.
Esposito, Luciana
Ettrich, Rudiger
Evans, Neil
Evers, Jochem
Eyras, Eduardo
Faeder, James R.
Faisal, Aldo A.
Faugeras, Olivier
Fearnhead, Paul

Featherstone, Carol
Fei, Zhangjun
Feig, Michael
Feil, Edward J.
Felts, Anthony
Feng, Jianfeng
Ferguson, Neil M.
Fernandez-Ballester, Gregorio
Fernandez-Recio, Juan
Fernando, Chrisantha
Ferrari, Matt
Ferron, François
Fink, J. L.
Finke, Christian
Fiser, Andras
Fiser, Jozsef
Fishelovitch, Dan
Fisher, Jasmin
Fisher, Michael
Fleischer, Frank
Fleischman, Sarel
Flower, Darren R.
Fodor, Anthony
Fong, Stephen S.
Fontanari, Jose Fernando
Foo, Jasmine
Forst, Christian
Foster, David
Foulkes, Nicholas S.
Fox, George E.
Fox, Thomas
Fraser, Hunter
Freemont, Paul S.
Friedman, Nir
Frishman, Dmitrij
Friz, Jonathan
Frost, Simon
Fry, Steven
Fryer, Helen
Fu, Bingmei
Furey, Terrence
Furman, Moran
Furman, Ora
Furney, Simon

Fusi, Stefano
Gütig, Robert
Gaasterland, Terry
Galdzicki, Michal
Gallivan, Justin
Galun, Eithan
Galvani, Alison P.
Ganusov, Vitaly V.
Garcia-Ojalvo, Jordi
Garrity, George
Gautheret, Daniel
Gavin, Anne-Claude
Gavrilets, Sergey
Ge, Hui
Gedeon, Tomas
George, Andrew
Georgiades, Pantelis
Gerhard, Klebe
Gerstein, George
Gerstein, Mark B.
Gerstner, Wulfram
Gfeller, David
Ghosh, Samik
Giddings, Morgan C.
Gielen, Stan
Gierasch, Lila
Giese, Martin A.
Gilchrist, Michael
Gilson, Michael
Ginsburg, Hagai
Gintis, Herbert
Girolami, Mark A.
Gloor, Greg B.
Gnanakaran, S.
Gohlke, Holger
Goldberg, Ilya G.
Goldberger, Ary L.
Golding, Ido
Goldman, Mark S.
Goldreich, Daniel
Goldstein, Raymond E.
Goldstein, Richard A.
Gollisch, Tim
Golomb, David
Golowasch, Jorge

Gomes, M Gabriela M.
Gomez, Shawn
Gonze, Didier
Goodhill, Geoffrey J.
Gordon, D. Benjamin
Gough, Julian
Goulian, Mark
Goyal, Sidhartha
Gräter, Frauke
Grabowecky, Marcia
Graham, Daniel
Gramada, Apostol
Grate, Leslie R.
Graw, Frederik
Gray, Jack
Gray, Jeffrey J.
Gregor, Thomas
Griffiths, Thomas
Grima, Ramon
Grishin, Nick
Gros, Claudius
Gross, Thilo
Grossman, Zvi
Gruen, Sonja
Gu, Charles
Guckenheimer, John M.
Guigon, Emmanuel
Guiot, Caterina
Gummer, Anthony
Gunawan, Rudiyanto
Guo, Chin-Lin
Gursoy, Attila
Gusfield, Dan
Gutkin, Boris S.
Haag, Juergen
Haas, Julie S.
Hachiya, Tsuyoshi
Hagen, Stephen
Hahn, Juergen
Halfon, Marc S.
Haliloglu, Turkan
Hall, Carol
Hall, David
Halloy, José
Hamer, Bojan

Hammell, Molly	Hofmeyr, Jan-Hendrik S.	Ionides, Ed
Hammer, Daniel A.	Hogan, Neville	Ioshikhes, Ilya
Hamosh, Ada	Hogeweg, Paulien	Iossifov, Ivan
Han, Lihui	Hohwy, Jakob	Irwin, John J
Hanage, William P.	Holden, James	Issa, Naoum
Handel, Andreas	Holderied, Marc	Iturria-Medina, Yasser
Hannenhalli, Sridhar	Holiday, Gemma	Izawa, Jun
Hansen, Niclas	Holm, Liisa	Izhikevich, Eugene M.
Harrow, Jennifer	Holmes, Ian	Jackson, Richard M.
Hartemink, Alexander J.	Holmgren, Arne	Jackson, Sophie
Haspel, Nurit	Holyoak, Keith	Jacob, Ute
Hasselmo, Michael E.	Honavar, Vasant	Jafri, Saleet
Hasty, Jeff	Honey, Christopher	Jain, Ajay
Hatzigeorgiou, Artemis	Honig, Barry	Janes, Kevin
Hatzimanikatis, Vassily	Hook, Vivian	Janga, Sarath C.
Haugh, Jason M.	Hopfield, John J.	Janin, Joel
Hayward, Steven	Hoppensteadt, Frank	Janke, Axel
He, Bin	Horesh, Yair	Janssen, Paul J.
Heermann, Dieter	Horovitz, Amnon	Jeka, John
Heinemann, Matthias	Horton, Paul	Jennings, Steven F.
Heinke, Dietmar	Horvath, Steve	Jernigan, Robert L.
Helmer-Citterich, Manuela	Houk, James C.	Jewett, Michael
Helms, Volkhard	House, Thomas	Jin, Yumi
Henson, Michael A.	Howard, Jonathon	Johnson, Frederick B.
Hermsen, Rutger	Howard, Mark	Johnson, Michael
Herrgard, Markus	Hsu, David	Johnson, Steve
Herzel, Hanspeter	Huang, Sui	Jones, Laura E.
Herzog, Michael H.	Huang, Xuhui	Jones, Steven J.
Herzog, Walter	Huber, Wolfgang	Jonsson, Henrik
Heymsfield, Steven B.	Hubler, Alfred	Jordan, I. King
Hibbs, Matthew A.	Hubmayr, Rolf	Josephson, RK
Higgins, Des G.	Huerta, Ramon	Jung, Peter
Hildebrand, Peter W.	Hughes, Timothy R.	Jurisica, Igor
Hildreth, Ellen	Hui, Raymond	König, Rainer
Hilfinger, Andreas	Humphreys, Glyn	Kaderali, Lars
Hilgetag, Claus C.	Humphries, Mark	Kaeberlein, Matt
Hill, James	Hurst, Laurence D.	Kaessmann, Henrik
Hill, Sean	Huson, Daniel H.	Kageyama, Ryoichiro
Hillis, David M.	Huttenhower, Curtis	Kahveci, Tamer
Hilser, Vincent J.	Huys, Quentin J.	Kaiser, Marcus
Hirano, Yoshinori	Hwang, Daehee	Kalinina, Olga V.
Hirschman, Lynette	Iglesias, Pablo A.	Kalisman, Nir
Hlavacek, William S.	Ikegami, Takashi	Kamei, Daniel
Hofer, Thomas	Indic, Premananda	Kamen, Amine
Hoffman, Michael	Ingalls, Brian	Kamermans, Maarten

Kamm, Roger D.	Kitzbichler, Manfred G.	Krukenberg, Kristin
Kaneko, Kunihiko	Klein, Stanley A.	Kruse, Karsten
Kannan, Natarajan	Klein, Teri	Krystek, Stanley R.
Kaplan, Tommy	Kleinschmidt, Andreas	Kubatko, Laura S.
Karniel, Amir	Kleinstein, Steven H.	Kudler, Grzegorz
Karpievitch, Yuliya V.	Klenerman, Paul	Kufareva, Irina
Kasischke, Karl A.	Klinke, David J.	Kuhlman, Brian
Kass, Robert	Klipp, Edda	Kuhlman, Thomas
Kasson, Peter	Kloczkowski, Andrzej	Kuhn, Michael
Katzenellenbogen, John	Klumpp, Stefan	Kuhn, Richard J.
Kay, Leslie	Knapp, Stefan	Kummer, Ursula
Kazic, Toni	Knight, Bruce	Kunz, Wolfram
Kaznessis, Yiannis N.	Knight, Rob	Kuo, Art
Keeney, Scott	Kobayashi, Ryota	Lacquaniti, Francesco
Keich, Uri	Kobilka, Brian	Lage Hansen, Kasper
Keles, Sunduz	Koelle, Katia	Lagergren, Jens
Kellis, Manolis	Koenig, Peter	Lages, Martin
Kelso, J.A. Scott	Kohlbacher, Oliver	Landy, Michael S.
Kemp, Melissa L.	Kolinski, Andrzej	Lane, Terran
Kenah, Eben	Kollmann, Markus	Lange, Markus
Kennedy, David	Kolmar, Harald	Langille, Morgan
Kern, Dorothee	Kolomeisky, Anatoly	Lapidus, Lisa
Kerr, Benjamin	Kondev, Jane	Laskowski, Roman A.
Kesmir, Can	Kondor, Risi	Latash, Mark
Keun, Hector	Koonin, Eugene V.	Latham, Peter E.
Khan, Amir	Korber, Bette T.	Laub, Michael T.
Kheradpour, Pouya	Kording, Konrad P.	Laurent, Louise C.
Khodursky, Arkady B.	Korkin, Dmitry	Lavery, Richard
Kidd, Jeffrey M.	Korona, Ryszard	Lawrence, Charles E.
Kidera, Akinori	Kosakovsky Pond, Sergei L.	Le Noble, Ferdinand
Kiebel, Stefan J.	Koulakov, Alex	Le Novère, Nicolas
Kiel, Christina	Kouyos, Roger D.	Leatherwood, Janet K.
Kihara, Daisuke	Kovacevic, Natasa	Lee, BK
Kilner, James	Kowald, Axel	Lee, Jae K.
Kim, Junhyong	Koyutürk, Mehmet	Lee, Eunjung
Kim, Kyung	Kozubek, Stanislav	Lee, Hyunju
Kim, Philip M.	Kra, Pauline	Lee, Jong Min
Kim, Seungchan	Krakauer, John	Lee, Keun Woo
Kingsford, Carl	Krallinger, Martin	Lee, Kyongbum
Kinnunen, Paavo	Kranczioch, Cornelia	Lee, Su-in
Kinzer-Ursem, Tamara	Krangel, Michael S.	Lee, Tai Sing
Kishony, Roy	Krauss, Scott	Lee, Tony
Kiss, Istvan	Kriener, Birgit	Legendre, Matthieu
Kiss, Tamas	Krishna, Mallela	Legenstein, Robert
Kitao, Akio	Kriwacki, Richard W.	Legewie, Stefan

Legrand, Judith
Lehman, Steven
Lehner, Ben
Leibold, Christian
Leitner, David
Leloup, Jean-Christophe
Lemey, Philippe
Lensink, Marc F.
Leon, Mike
Lerchner, Alexander
Leslie, Christina
Lespinet, Olivier
Levchenko, Andre
Levin, Matthew
Levine, Erel
Levine, Herbert
Levo, Michal
Levy, Koby
Lewis, Suzanna
Lewitter, Fran
Lezon, Timothy R.
Li, Gene-Wei
Li, Heng
Li, Hongzhe
Li, Weizhong
Li, Yue
Liang, Jie
Liang, Liming
Liao, Ben-Yang
Liao, Li
Liberles, David
Lim, Roderick
Lin, David
Lindahl, Erik
Lindemann, J. P.
Lindner, Benjamin
Linial, Michal
Linkenkaer-Hansen, Klaus
Linster, Christiane
Lion, Sébastien
Lipan, Ovidiu
Lipsitch, Marc
Litvak, Vladimir
Liu, Jun S.
Liu, Xiaole

Lively, Curt
LiWang, Andy
Lloyd-Smith, James
Loeb, Gerald
Logie, Colin
Logsdon, Benjamin
Lomize, Andrei
Loomis, Jack
Lorenceau, Jean
Lotto, R. B.
Louzoun, Yoram
Low, David
Lundkvist, Gabriella
Lunter, Gerton
Luo, Ray
Lupas, Andrei N.
Lussier, Yves
Müller, Viktor
Münch, Richard
Ma, Jianpeng
Ma, Wei Ji
Ma'ayan, Avi
Mac Gabhann, Feilim
Macallan, Derek C.
MacAlpine, David M.
MacDonald, Colin
Machens, Christian K.
Madzvamuse, Anotida
Maex, Reinoud
Mager, Dixie L.
Maggelakis, Sophia
Magwene, Paul M.
Mahadevan, Radhakrishnan
Mahaffy, Joseph
Maini, Philip
Makarov, Dmitrii
Maley, Carlo C.
Mancilla, Jaime
Mandel-Gutfreund, Yael
Manrubia, Susanna C.
Maranas, Costas D.
Maraziotis, Ioannis
Marcovitch, Stuart
Margulies, Susan S.

Marinazzo, Daniele
Maritan, Amos
Markel, Scott
Markov, Peter
Markowetz, Florian
Marks, Debora
Martin, Kevan A.C. A.
Martinez, Salvador
Martins dos Santos, Vitor A.P.
Masel, Joanna
Maslov, Sergei
Masotti, Andrea
Mata, Juan
Mattingly, Carolyn
Mau, Bob
Mayr, Christine
McArdle, Craig
McClendon, Chris
McCluskey, Connell
McElwain, Donald L.
McIntosh, A. Randy
McIntosh, Martin W.
McKee, Sean
McMillen, Dave
Meeter, Martijn
Mehr, Ramit
Mehring, Carsten
Mehta, Pankaj
Meier-Schellersheim, Martin
Meijer, Johanna H.
Meinhardt, Hans
Meinnel, Thierry
Meister, Gunter
Meister, Markus
Mendes, Pedro
Meredith, Steve
Merfeld, Daniel M.
Meroueh, Samy
Mestres, Jordi
Metzler, Ralf
Meyer-Hermann, Michael E.
Meyers, Lauren A.

Mi, Huaiyu
Micheletti, Cristian
Michnick, Stephen W.
Miedema, Frank
Miller, Christopher S.
Miller, Joel C.
Miller, John
Minai, Ali
Minayev, Pavlo
Mirny, Leonid A.
Mitra, Robi D.
Mitrophanov, Alexander Y.
Mittler, John E.
Miyano, Satoru
Miyazawa, Sanzo
Mobley, David
Mogilner, Alex
Mohr, Johannes
Molenaar, Douwe
Monéger, Françoise
Mongillo, Gianluigi
Moore, Cristopher
Moran, Rosalyn J.
Moreau, Yves
Morgenstern, Burkhard
Morowitz, Harold J.
Morozov, Alexandre
Morris, Quaid
Morrison, Abigail
Morse, Randy
Mortenson, Paul
Mortimer, Duncan
Moya, Andres
Moyer, Jason
Muchmore, Steven
Muckli, Lars
Mukherjee, Sayan
Muller, Eilif
Mullins, James I.
Mulloney, Brian
Munsky, Brian
Murphy, Coleen T.
Murphy, Robert
Murray, Douglas B.
Murray, Richard

Murzin, Alexey
Mustonen, Ville
Myers, Chad L.
Myers, Christopher R.
Myers, Donald
Nadal, Jean-Pierre
Naef, Felix
Nagano, Nozomi
Nagaraj, Nagathihalli S.
Nakai, Kenta
Nakhleh, Luay
Nash, Piers
Neduva, Victor
Neiman, Maurine
Nelken, Israel
Neri, Peter
Newman, Mark
Newman, Stuart
Newman, Tim
Nicolas, Alain G.
Nicolas, Alice
Nielsen, Cydney
Nielsen, Jens
Nielsen, Jens Erik
Nielsen, Morten
Nikaido, Hiroshi
Nilges, Michael
Nilssen, Lennart
Nimrod, Guy
Nirenberg, Sheila
Nix, David
Noe, Frank
Noppeney, Uta
Noskov, Sergei
Notebaart, Richard A.
Novo, Francisco J.
Nussinov, Ruth
O'Doherty, John
O'Donoghue, Sean I.
O'Rourke, Brian
Obermayer, Klaus
Odde, David
Ofran, Yanay
Ohler, Uwe
Ohtani, Naoko

Okamura, Hitoshi
Okita, Thomas
Okuno, Yasushi
Olbrich, Eckehard
Oliva, Aude
Oliva, Baldomero
Olshausen, Bruno
Oltvai, Zoltan N.
Olufsen, Mette
Omenn, Gilbert S.
Orengo, Christine A.
Oresic, Matej
Osterman, Andrei
Othmer, Hans
Otto, Sarah P.
Owen, Markus
Oyama, Masaaki
Paci, Emanuele
Paige, Gary
Palomero-Gallagher, Nicola
Palsson, Bernhard &.
Panchenko, Anna R
Panek, Josef
Panetta, Carl
Panfilov, Sasha
Panzeri, Stefano
Papin, Jason A.
Papke, R. Thane
Papoian, Garegin
Papp, Balázs
Pappu, Rohit V.
Park, Peter
Parkinson, John
Pastore, Annalisa
Paten, Benedict
Paternostro, Giovanni
Pathy, Nim
Patterson, Nick
Pawelzik, Klaus R.
Pazos, Florencio
Pe'er, Dana
Pe'er, Itsik
Pedersen, Michael
Peirce, Shayn M.
Pellegrini, Matteo

Peng, Hanchuan
Perahia, David
Perc, Matjaz
Perelson, Alan S.
Pereverzeva, Maria
Perkins, Theodore J.
Perrakis, Anastassis
Pessiglione, Mathias
Peterka, Robert
Peters, Bjoern
Peterson, Carsten
Pfister, Jean-Pascal
Pfurtscheller, Gert
Piano, Fabio
Piermarocchi, Carlo
Pillow, Jonathan
Pilyugin, Sergei S.
Pitera, Jed
Planes, Francisco J.
Plaxco, Kevin
Plesser, Hans Ekkehard
Plotkin, Joshua B.
Poets, Christian
Pollard, Daniel
Polyak, Kornelia
Pomerening, Joseph R.
Ponce Dawson, Silvina
Ponting, Chris P.
Poo, Mu-ming
Poole, Leslie
Popel, Aleksander S.
Porto, Markus
Posas, Francesc
Potapov, Vladimir
Pouget, Alex
Pourbohloul, Babak
Preissner, Robert
Prescott, Steve
Priami, Corrado
Price, Nathan D.
Prlic, Andreas
Przytycka, Teresa M.
Punta, Marco
Punta, Marco
Pybus, Oliver G.

Qi, Feng
Qi, Yanjun
Qian, Hong
Qian, Jiang
Qutub, Amina A.
Rabadan, Raul
Rabinovich, Mikhail I.
Rader, Andrew
Radivojac, Predrag
Raes, Jeroen
Raghava, Gajendra P.
Raghavachari, Sridhar
Rajewsky, Nikolaus
Rajwa, Bartek
Ramamoorthy, A
Raman, Indira
Rambaut, Andrew
Ramoni, Marco F.
Rando, Oliver J.
Ranganathan, Rama
Rangel, Antonio
Rankin, Daniel J.
Rao, Christopher
Rarey, Matthias
Rasko, David
Rasmusson, Randall
Rayner, Keith
Rebholz-Schuhmann, Dietrich
Recker, Mario
Redish, A David
Reed, Jennifer
Reeves, Adam
Regoes, Roland R.
Reinkensmeyer, David
Relman, David A.
Reluga, Timothy
Remez, Robert
Renart, Alfonso
Reynolds, Sheila M.
Rhodes, Paul
Ribeiro, Ruy M.
Riester, Markus
Rigoutsos, Isidore
Riley, Steven

Rind, Claire
Rinn, John
Ritchie, David
Robert, Gentleman
Roberts, Eugene
Robertson, David L.
Robin, Stephane
Robinson, Hugh P.
Robinson, Peter
Rocco, Andrea
Rocha, Eduardo P.
Rodriguez-Caso, Carlos
Rodriguez-Esteban, Raul
Roeder, Ingo
Rogers, Simon
Rohani, Pejman
Rohs, Remo
Ronquist, Fredrik
Roper, Peter
Rosa, Angelo
Rosa, Marcello G.
Rose, Dominic
Ross, Elliott M.
Rossini, Paolo M.
Roth, Arnd
Roth, Fritz
Rouchka, Eric C.
Roudi, Yasser
Roussel, Marc R.
Roux, Aurelien
Rouzine, Igor M.
Rowicka, Malgorzata
Roy, Krishnendu
Rozen, Steve
Ruan, Jianhua
Rubin, Jonathan
Ruediger, Stefan
Ruffier, Frank
Rundell, Ann
Ruppin, Eytan
Rushworth, Matthew F. S.
Russell, Brenda
Russell, Colin A.
Rux, John
Ryan, Margaret

Ryu, William
Sa, Rui C.
Saez-Rodriguez, Julio
Saftenku, Elena
Sajda, Paul
Salama, Guy
Salathe, Marcel
Salis, Howard
Salsbury, Jr, Freddie
Salvador, Armindo
Samudrala, Ram
Sanchez, Roberto
Sandaa, Ruth-Ann
Sandberg, Rickard
Sander, Chris
Sandercock, Thomas
Sandstede, Bjorn
Sanejouand, Yves-Henri
Sansone, Susanna-Assunta
Santamaria, Fidel
Sarkar, Casim A.
Sarkar, Neil
Sasikumar, Roschen
Saucerman, Jeffrey J.
Sauer, Uwe
Sauna, Zuben E.
Sauro, Herbert M.
Sbalzarini, Ivo
Schäffer, Alejandro A.
Schöler, Hans
Schaap, Pauline
Schaffer, David V.
Scheidt, Robert
Schimansky-Geier, Lutz
Schlicker, Andreas
Schmucker, Dietmar
Schneidman, Elad
Schnell, Santiago
Schnitzer, Mark
Schroeder, Charles
Schueler-Furman, Ora
Schuler, Maik
Schultz-Hector, Susanne
Schulze, Waltraud X.
Schuster, Stefan
Schwartz, Andrew B.
Schwede, Torsten
Schweighofer, Nicolas
Schymkowitz, Joost
Sciabola, Simone
Secomb, Timothy W.
Segal, Eran
Seger, Rony
Segre, Daniel
Seidler, Rachel
Sejnowski, Terrence J.
Semple, Colin
Senger, Ryan S.
Serre, Thomas
Seshasayee, Aswin Sai Narain
Shadlen, Michael N.
Shafer, Robert W.
Shakhnovich, Eugene I.
Shamir, Ron
Shamma, Shihab A.
Shamovsky, Ilya
Shapiro, Mark
Sharan, Roded
Sharp, Andrew J.
Shatkay, Hagit
Shaw, Richard
Shea, Joan-Emma
Shea-Brown, Eric
Shearer, Francesca
Shen-Orr, Shai
Shendure, Jay
Shi, Yufang
Shimizu, Tom
Shimkets, Lawrence J.
Shimoni, Yishai
Shirts, Michael
Shlomi, Tomer
Shomron, Noam
Shouval, Harel Z.
Shraiman, Boris I.
Shvartsman, Stanislav
Sidhu, Sachdev
Siegal, Mark
Siegel, Dave
Siegel, Markus
Simmons, James
Simon, Giszter
Simonson, Thomas
Simpson, Michael L.
Singh, Mona
Sinha, Rileen
Sinha, Saurabh
Siomi, Haruhiko
Sirota, Anton
Sjölander, Kimmen
Skudlarski, Pawel
Slatkin, Monty
Slonim, Donna K.
Sluis-Cremer, Nicolas
Smallwood, Rod
Smeets, Jeroen
Smith, Barry
Smith, David L.
Smith, Gavin
Smith, Linda
Smith, Lucian
Smith, Maurice A.
Smith, Nicolas P.
Smith, Richard S.
Sneppen, Kim
Snoeyink, Jack
Solovei, Irina
Solovyev, Victor
Soltani, Alireza
Song, Wenchao
Sorribas, Albert
Souman, Jan
Sourjik, Victor
Soyer, Orkun S.
Speakman, John
Spicer, Leonard
Spormann, Alfred M.
Sporns, Olaf
Spratling, Michael
Sprekeler, Henning
Srinivasan, Balaji S.
Srinivasan, Manoj
Srivastava, Ranjan
Stam, Cornelis J.

Stamatoyannopoulos, John A.
Starmer, Frank
Stefanovic, Darko
Stekel, Dov J.
Stepanova, Maria
Stephan, Klaas E.
Steuer, Ralf
Stevens, Rick L.
Stiefel, Klaus M.
Stoma, Szymon
Stone, Eric A.
Stopfer, Mark
Stormo, Gary D.
Stripling, Jeffrey
Strowbridge, Ben
Stuart, Josh
Subramaniam, Shankar
Subramanian, Sri K.
Suki, Bela
Sumpter, David J.
Sun, Fengzhu
Sun, Zhirong
Sunkar, Ramanjulu
Surmeier, D. J.
Swain, Peter
Swint-Kruse, Liskin
Szathmáry, Eörs
Szatmary, Botond
Sze, Sing-Hoi
Tabach, Yuval
Tajkhorshid, Emad
Takahata, Naoyuki
Talathi, Sachin
Tamames, Javier
Tanaka, Mark M.
Tang, Chao
Tang, Haixu
Tans, Sander J.
Tarnita, Corina E.
Taylor, Adam
Taylor, James
Teixeira, Miguel
Ten Wolde, Pieter
Tenaillon, Olivier
Tenson, Tanel
Teramae, Jun-nosuke
Tetzlaff, Tom
Teusink, Bas
Theobald, Douglas L.
Thierry, Bernard
Thirumalai, Dev
Thomas, Wendy E.
Thompson, William
Thomson, J. M.
Thoroughman, Kurt A.
Thorpe, Simon J.
Tiana, Guido
Tidor, Bruce
Tiesinga, Paul
Tiffin, Nicki
Tildesley, Michael J.
Timme, Marc
Tirosh, Itay
Tolkunov, Denis
Tolstorukov, Michael
Tompa, Martin
Toroczkai, Zoltan
Torrents, David
Tosatto, Silvio C.
Toyoizumi, Taro
Trajanoski, Zlatko
Traulsen, Arne
Travers, Andrew A.
Trifonov, Edward N.
Trotter, Matthew
Trovato, Antonio
Trujillo-Barreto, Nelson J.
Tsai, Gavin
Tschumperlin, Daniel J.
Tsotsos, John
Tsunoda, Tatsuhiko
Tu, Yuhai
Tuler, Tamir
Turner, Ray
Tyson, John J.
Ulitsky, Igor
Ullmann, Matthias
Umulis, David
Unger, Ron
Upton, Chris
Urban, Nathaniel N.
Uversky, Vladimir N.
Vaidehi, Nagarajan
Vajda, Sandor
Vaknin, Ady
Valencia, Alfonso
Van Beers, Rob
Van der Giessen, Erik
Van der Horst, Gijsbertus T.
Van der Spek, Peter
Van der Vaart, Arjan
Van Nimwegen, Erik
Van Oijen, Antoine M.
Van Ooyen, Arjen
Van Riel, Natal
Van Rossum, Mark C.
Van Swinderen, Bruno
Van-Vreeswijk, Carl
Varner, Jeffrey D.
Vavylonis, Dimitrios
Vendelin, Marko
Vendruscolo, Michele
Veng-Pedersen, Peter
Venturi, Venessa
Vera-Licona, Paola
Veretnik, Stella
Verschure, Paul F.
Verspoor, Karin
Vert, Jean-Philippe M.
Vicsek, Tamas
Vihinen, Mauno
Vingron, Martin
Vinnakota, Kalyan C.
Vishveshwara, Saraswathi
Vita, Randi
Vitek, Olga
Vogels, Tim P.
Voigt, Christopher A.
Voit, Eberhard
Voliotis, Margaritis
Volz, Erik
Von Mering, Christian
Vriend, Gert
Wörgötter, Florentin

Wackett, Lawrence P.	Westhead, David R.	Xuan, Zhenyu
Wade, Rebecca C.	Wetzel, Ronald	Yakhini, Zohar
Wagner, Günter P.	White, Peter	Yamaguchi, Yoko
Wainberg, Mark	Wierling, Christoph	Yandell, Brian S.
Waldispuhl, Jerome	Wiggins, Chris	Yang, Feng
Walhout, Marian	Wilhelm, Thomas	Yang, Wei
Wall, Dennis P.	Wilke, Claus O.	Yarmush, Martin
Wall, Michael E.	Williams, Stephen	Yates, Andrew
Wallinga, Jacco	Willner, Dana	Ye, Ping
Wallner, Björn	Wilson, Marcus	Yeates, Todd O.
Wallraven, Christian	Wilson, William J.	Yi, Soojin V.
Walton, Mark	Wingreen, Ned S.	Yin, John
Wand, Joshua	Winslow, Raimond	Yin, Shuangye
Wang, Edwin	Winther, Ole	York, Darrin
Wang, Jian	Wirtz, Denis	Yosef, Nir
Wang, Jin	Wiskott, Laurenz	Yoshida, Wako
Wang, Kai	Wodak, Shoshana	You, Lingchong
Wang, May	Wodarz, Dominik	Young, Jasmin
Wang, Ting	Wojtowicz, Damian	Yu, Hong
Wang, Wei	Wolgemuth, Charles W.	Yu, Jun
Wang, Xiao-Jing	Wong, Chung	Yuan, Yaowu
Wang, Xiaowei	Wong, Stephen T.	Yue, Hong
Wang, Yi	Wood, Graham	Yugi, Katsuyuki
Wang, Yusu	Wood, John	Zacharias, Martin
Wang, Zhi	Woodgett, James	Zaks, Michael
Ward, Lawrence	Woolf, Peter J.	Zallen, Jennifer
Warwicker, Jim	Woolf, Thomas	Zaman, Muhammad
Warzecha, Anne-Kathrin	Wootton, J. Timothy	Zandstra, Peter
Washburn, Michael	Wouters, Bradly	Zavolan, Mihaela
Watson, Richard A.	Wrana, Jeffrey L.	Zenker, Sven
Waxman, Stephen G.	Wray, Gregory A.	Zhang, Jianzhi
Weaver, Alissa M	Wu, Fan	Zhang, Lixin
Webb, Alex	Wu, Lani F.	Zhang, Weixiong
Webb, Barbara	Wu, Martin	Zhang, Xiang-Sun
Wei, Guanghong	Wu, Song	Zhang, Xuegong
Wei, Liping	Wylie, Scott	Zhang, Yang
Weikl, Thomas	Xenarios, Ioannis	Zhang, Zhaolei
Weinstock, George M.	Xia, Di	Zhao, Keji
Weiss, Yair	Xia, Yu	Zhao, Ting
Weitzenfeld, Alfredo	Xiao, Xinshu	Zhao, Zhongming
Welch, Roy D.	Xie, Lei	Zhaopiing, Li
Wennberg, Bernt	Xie, Li	Zheng, Deyou
Wennekers, Thomas	Xing, Yi	Zheng, Jie
Wernisch, Lorenz	Xu, Dong	Zheng, Yun
Wessel, Niels	Xu, Ying	Zhong, Sheng

Zhong, Weiwei
Zhou, Huan-Xiang
Zhou, Ruhong
Zhou, Xianghong J.
Zhou, Xuefeng
Zhou, Yaoqi
Zhu, Cheng
Zhu, Jian-Kang
Zhurkin, Victor
Zucker, Steve
Zunger, Yonatan
Zupan, Blaz
